# A Rare Case of Atypical Hemolytic Uremic Syndrome (aHUS) Precipitated by Dengue and the Treatment Landscape in Singapore

**DOI:** 10.7759/cureus.58731

**Published:** 2024-04-22

**Authors:** Yan Chin Tan, Esmeralda Chi Yuan Teo, Heng Joo Ng

**Affiliations:** 1 Haematology, Sengkang General Hospital, Sengkang, SGP; 2 Haematology, Singapore General Hospital, Bukit Merah, SGP

**Keywords:** renal failure, complement factor h related protein, plasma exchange therapy, eculizumab, thrombotic thrombocytopenic purpura (ttp)-like syndrome, dengue fever/complications, thrombocytopenia, hemolytic anaemia, thrombotic microangiopathy (tma), atypical hemolytic uremic syndrome

## Abstract

Atypical hemolytic uremic syndrome (aHUS) is a rare disease caused by uncontrolled complement activation due to complement dysregulation. It is often triggered by precipitating events such as infections, inflammation, pregnancy, or medications. Dengue, an endemic viral infection in Southeast Asia, can activate the complement pathway, thereby triggering aHUS in genetically susceptible individuals. Here, we present the case of a 33-year-old male who presented with Dengue fever and subsequently developed aHUS. Plasma exchange (PLEX) successfully normalized his neurological status and hematological parameters. Although his renal function improved, it failed to normalize. Eculizumab, a monoclonal antibody that inhibits C5, was administered for a total of six months. The treatment was successfully discontinued without evidence of relapse after six months of follow-up. This case report demonstrates the safety of discontinuing eculizumab in patients who do not possess pathogenic mutations or variants in complement factors.

## Introduction

Atypical hemolytic uremic syndrome (aHUS) is an ultra-rare disease with a prevalence of 0.29-1.9 per million population annually [1]. It is characterized by thrombotic microangiopathy with end-organ damage due to complement dysregulation [2]. Eculizumab, the treatment of choice for aHUS, was once thought to require lifelong administration, but this comes with a prohibitive financial cost. In recent years, more evidence has surfaced supporting a more cost-effective, time-limited use of Eculizumab in low-risk patients. Here, we report a case of aHUS precipitated by a dengue infection, successfully treated with a finite duration of Eculizumab.

## Case presentation

A 33-year-old male was admitted for dengue fever. He presented with fever and myalgia and was found to be profoundly thrombocytopenic. Upon admission, his hemoglobin was 15.4 g/dL (normal: 14-16.5 g/dL), platelet count was 20 x 10^9/L (normal: 150-450 x 10^9/L), and creatinine was 200 µmol/L (normal: 59-104 µmol/L). His dengue diagnosis was confirmed by positive dengue antigen testing, i.e., nonstructural protein-1 (NS-1), and positive dengue serology tests, namely immunoglobulin M (IgM) and IgG.

**Figure 1:**
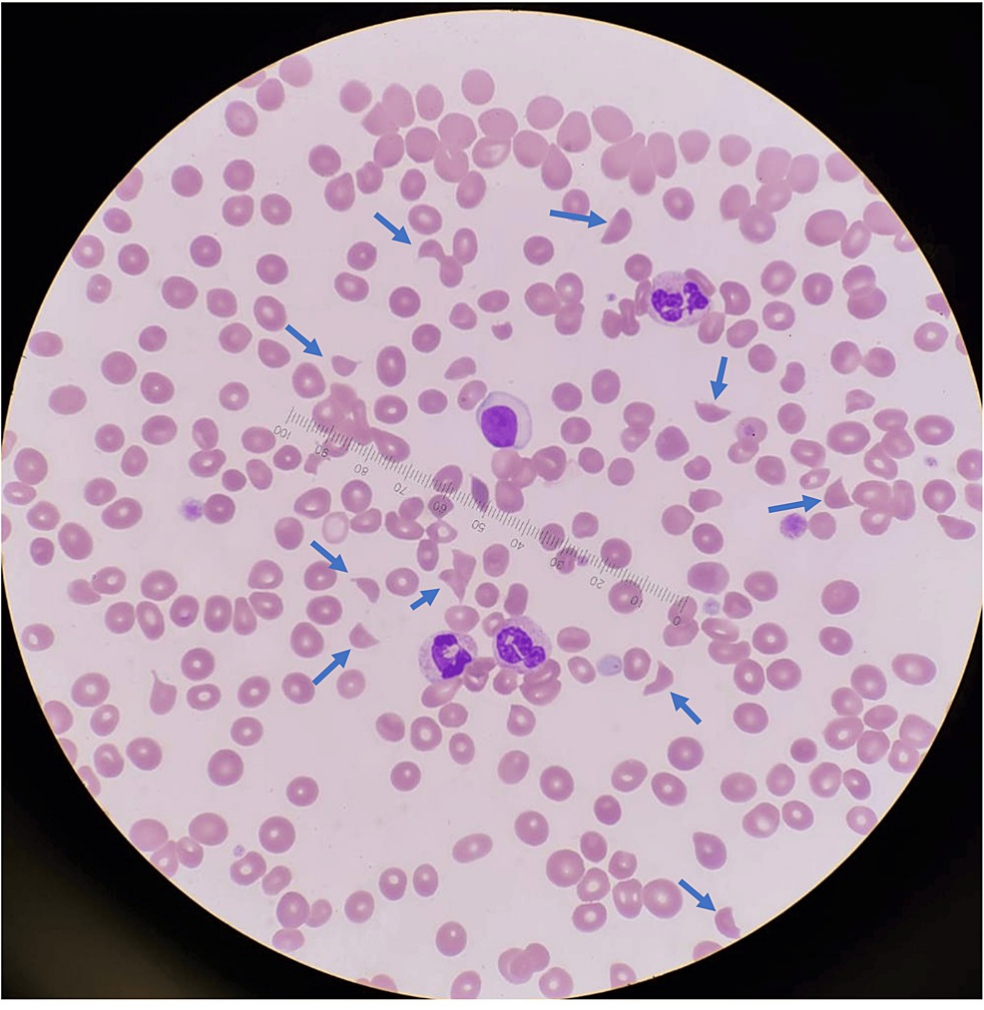
Peripheral blood film at diagnosis showing markedly elevated schistocytes (10 per high-power field, indicated by blue arrows) and thrombocytopenia, observed under 100x oil immersion.

On the second day of admission, he developed massive upper gastrointestinal bleeding secondary to a gastric ulcer. He required intubation for airway protection, endoscopic hemostasis, and a transfusion. His clinical condition improved after hemostasis was achieved. However, on the fifth day of admission, he developed an altered mental state, worsening thrombocytopenia, renal function, and Coombs-negative hemolytic anemia. His hemoglobin dropped to 6.3 g/dL, platelets to 31 x 10^9/L, and creatinine increased to 400 µmol/L. A hemolysis screen was positive: elevated bilirubin 40 µmol/L (normal <21 µmol/L), low haptoglobin <0.1 g/L (normal: 0.3-2.0 g/L), raised lactate dehydrogenase (LDH) of 554 U/L (normal 222-454 U/L), and a blood film showing numerous schistocytes as shown in Figure 1.

He was initially treated as presumptive thrombotic thrombocytopenic purpura (TTP). Plasma exchange (PLEX) was promptly initiated. Two days after starting PLEX, his confusion resolved, and his platelet count improved to 53 x 10^9/L without requiring further transfusions. As his ADAMTS13 level subsequently returned normal at 47%, TTP was ruled out. Shiga toxin-producing E. coli (STEC) infection was also excluded due to a negative stool culture. Therefore, a diagnosis of aHUS triggered by dengue fever was made. PLEX was continued daily, and the platelet count normalized on day 12 of PLEX. The frequency of PLEX was reduced to every other day upon platelet count normalization, eventually minimizing to twice a week within five weeks from initiation. Despite a rapid hematological response, his renal response lagged, with creatinine returning to pre-PLEX levels only four weeks after PLEX (creatinine 150 µmol/L at steady state). Eculizumab was considered, but due to its high cost, we opted for maintenance PLEX. After 3.5 months on PLEX, he developed disseminated Mycobacterium abscessus septicemia from line sepsis, requiring admission for prolonged combination antibiotics. His aHUS genetic mutation panel detected a heterozygous variant of uncertain significance in the complement factor H related 3 (CFHR3) gene, i.e., CFHR3c.115C>T (p.Arg39Cys). This missense mutation is currently not known to be pathogenic. Eculizumab was initiated, and PLEX was discontinued simultaneously. There has been no clinical or laboratory evidence of relapse 6 months after Eculizumab discontinuation.

## Discussion

aHUS primarily affects the blood and kidneys, with more than 50% of patients progressing to end-stage renal disease (ESRD) [3]. It also impacts other systems, including neurological (48%), cardiovascular (43%), gastrointestinal (30%), and the pulmonary and ocular systems. Symptoms are variable and nonspecific, making diagnosis challenging [3].

Viral infections such as dengue activate complement pathways and can trigger aHUS in genetically susceptible individuals [4]. The case likely involves secondary dengue infection, as both dengue IgG and IgM serologies were positive. Dengue has four serotypes; being infected with one serotype does not confer immunity to the others. Secondary dengue infection occurs when an individual is infected with another serotype, often resulting in more severe clinical manifestations due to overwhelming complement activation, particularly in the alternative pathway [5]. In our patient, although the heterozygous variant detected in the CFH gene was not known to be pathogenic based on current literature, he could still be genetically susceptible (first hit). Dengue could have been the second hit event, causing unopposed complement activation and the development of aHUS.

The case met all the laboratory diagnostic criteria for aHUS adopted by most trials [6, 7]. Firstly, thrombotic microangiopathy (TMA) was evident, as shown by raised LDH, low haptoglobin, thrombocytopenia, and schistocytes (Figure 1). Schistocytes in the blood film support a diagnosis of TMA but are not required if the overall clinical picture is consistent with TMA [7]. Secondly, significant renal impairment was observed. Thirdly, aHUS mimicries such as STEC-related HUS and TTP were ruled out.

Although most guidelines recommend screening for CFH, Membrane Cofactor Protein (MCP/CD46), Complement Factor I (CFI), Complement factor B (CFB), Complement 3 (C3), Thrombomodulin (THBD), and CFH antibody to confirm complement pathway dysregulation [4], we did not perform any of these tests as they are unavailable in Singapore (except for C3). Furthermore, quantitatively normal CF levels do not reflect functionality [4]. Only 22% of patients harboring CFH mutations have low CFH levels, while 37% have normal levels [4]. Establishing a normal range for CFH and CFI is challenging due to wide variations across different age groups [4]. Most importantly, like ADAMTS13, these tests are affected by PLEX and must strictly be collected prior to the initiation of PLEX.

Genetic mutation studies are also recommended as part of the aHUS workup [4]. More than 50% of patients with aHUS carry genetic mutations in the complement system, with CFH mutation being the most common (24%), followed by MCP (7%), CFI, C3, and THBD (3-4%) [4]. Detection of heterozygous/homozygous pathogenic variants confirms the diagnosis of aHUS, although detection of non-pathogenic variants, like in our patient, does not rule out aHUS. Genetic mutation studies also help prognosticate, determine treatment duration, and predict response to PLEX. For example, MCP (CD46) mutations confer the most favorable renal outcome and mortality benefit, whereas CFH or THBD mutations are associated with the worst renal transplant outcomes. PLEX induces remission in 55-80% of episodes in patients with autoantibody H, CFH, C3, and THBD mutations, but patients with CFI mutations are poor responders [2]. PLEX replaces non-membrane-bound plasma proteins (e.g., CFH, C3) and removes Antibody H. This explains why our case with the CFHR3 variant responded well to PLEX.

However, long-term follow-up showed that 78% of patients had either died or developed ESRD with long-term PLEX [3]. Therefore, while PLEX is effective as an initial treatment, it should not be utilized indefinitely for aHUS.

Eculizumab is a monoclonal antibody that blocks C5 cleavage, thus preventing the formation of the proinflammatory peptide C5a and the C5b-9 membrane attack complex, which are central to the pathophysiology of aHUS [6]. It improves renal function and normalizes hematological parameters in 76% and 88% of patients, respectively [6]. Despite its effectiveness, Eculizumab has not been widely used due to its high cost. It was initially marketed as a lifelong treatment. Fortunately, multiple studies published recently have shown that discontinuing Eculizumab in patients without pathogenic variants, after a finite duration of 3-6 months, results in a low relapse rate, averaging 3.5% [8, 9]. This has allowed for significant cost savings and improved the rate of usage of Eculizumab. As our patient’s CFH3 variant is not currently classified as a known pathogenic mutation/variant, funding for six months of Eculizumab use was approved. Although he responded well neurologically and hematologically, his renal function failed to normalize despite Eculizumab. This could be due to the delay in initiating Eculizumab because of financial constraints. Walle JV et al. in their post hoc analysis showed a better renal outcome if Eculizumab can be started within seven days of diagnosis [10].

## Conclusions

Dengue can trigger aHUS in genetically susceptible individuals. While PLEX is an effective initial treatment in the non-CFI mutated group, it has failed to show long-term advantages. Compared to PLEX, Eculizumab offers superior renal outcomes and mortality benefits. It should be started early, ideally within seven days of diagnosis, and continued for a minimum of 3-6 months in patients without pathogenic variant mutations. This treatment approach saves costs without compromising its effectiveness.
